# Factors associated with delay in seeking care for breast symptoms

**DOI:** 10.1186/s12905-022-01898-5

**Published:** 2022-07-27

**Authors:** Jien Yen Soh, Maya Mazuwin Yahya, Norsa’adah Bachok, Wan Zainira Wan Zain, Michael Pak-Kai Wong, Zaidi Zakaria, Siti Rahmah Hashim Isa Merican, Mohd Nizam Md Hashim, Wan Mokhzani Wan Mokhter, Rosnelifaizur Ramely, Andee Dzulkarnaen Zakaria, Ikhwan Sani Mohamad

**Affiliations:** 1 Department of Surgery, Hospital Universiti Sains Malaysia, Universiti Sains Malaysia, 16150 Kubang Kerian, Kelantan Malaysia; 2 Unit of Biostatistics and Research Methodology, School of Medical Sciences, Universiti Sains Malaysia, 16150 Kubang Kerian, Kelantan Malaysia; 3 Breast Awareness and Research Unit (BestARi), Hospital Universiti Sains Malaysia, Kubang Kerian, Kelantan, Malaysia; 4 School of Medical Sciences, Universiti Sains Malaysia, Kubang Kerian, Malaysia

**Keywords:** Breast Neoplasms, Women, Education, Community

## Abstract

**Background:**

Breast cancer is the most common cancer in women worldwide. Early detection and intervention are associated with better prognosis and survival. The study aim was to investigate the factors associated with delayed presentation among women with breast symptoms.

**Methods:**

After ethics approval, a cross-sectional study was conducted from January to October 2020 in women with new breast cancer symptoms at their first visit to our clinic. The “Delayed Presentation” questionnaires in the Malay language were used and distributed among the participants. Demographic data and presentation time were recorded. Presentation time was defined as the duration of symptoms prior to visiting any health care facilities. Respondents with presentation times > 90 days comprised the delayed group. The potential factors associated with the delayed presentation were analyzed using cross-tabulation and multiple logistic regression.

**Results:**

There were 106 respondents to the questionnaire, with a mean age of 34.0 (SD: 11.2) years, and 73.6% (n = 78) were < 39 years old. A total of 35.8% (n = 38) visited the local government clinic first and only 28.3% (n = 30) came to the BestARi clinic directly. The reasons for presentation were a palpable breast lump on breast self-examination (75.5%, n = 80), mastalgia (15.1%, n = 16), nipple discharge (5.7%, n = 6), skin changes (0.9%, n = 1), and others (2.8%, n = 3). Among the respondents, 10.4% (n = 11) had alternative treatments prior to presentation to a hospital. The mean presentation time was 98.9 (SD: 323.7) days. Most of the participants (61.3%, n = 65) presented to us within 1 month. The delayed presentation group accounted for 19.8% (n = 21) of the respondents. The factor that was significantly associated with delayed presentation was the participants’ perception of symptoms as not dangerous (adjusted OR 3.05, 95% CI 1.11, 8.38).

**Conclusions:**

The percentage of delayed presentations among our patients was lower than the percentage reported in a previous study. Interpretation of a symptom as harmless by the respondent was the only factor significantly associated with delayed presentation.

**Supplementary Information:**

The online version contains supplementary material available at 10.1186/s12905-022-01898-5.

## Background

Breast cancer is the most common and deadliest cancer in women worldwide [1, 2]. In high-resource countries, the incidence and mortality rates of breast cancer have been declining, whereas in low-resource countries, the incidence and mortality rates have been increasing because of the differences in the access to breast cancer early detection [2]. In Malaysia, breast cancer accounts for 19% of all cancers [3]. The incidence of female breast cancer registered in the Malaysian National Cancer registry report has been increasing from 18,206 in 2007–2011 to 21,634 in 2012–2016, accounting for 34.1% of all cancers among females [3]. The incidence of breast cancer in Malaysia is expected to increase because of improving life expectancy, better socioeconomic status, and changes in lifestyles [4].

Delay in seeking examinations of breast symptoms is a significant problem [5] associated with a lower breast cancer survival rate [6]. It is important to provide awareness to communities that any breast symptoms should prompt early medical attention because they potentially could indicate breast cancer. A high percentage of breast symptoms turn out to be malignant. A study in London of 692 women who presented to the Breast Clinic with breast symptoms found that 87 (12.6%) of the women had a diagnosis of breast cancer [5].

The study aim was to investigate the potential factors associated with delay in seeking treatment among patients with breast symptoms who consulted with the Breast Cancer Awareness & Research Unit (BestARi) clinic, Hospital Universiti Sains Malaysia, Kubang Kerian, Kelantan.

## Methods

This was a cross-sectional study conducted at the BestARi clinic, Hospital Universiti Sains Malaysia, Kubang Kerian, Kelantan, from January to October 2020. The patients were women aged > 18 years with new breast symptoms at the first visit to the BestARi clinic who were invited to participate in this questionnaire study. Patients referred from other primary care centers or with a previous history of breast diseases (benign or cancer) who had new breast symptoms were also included. Patients who exhibited cognitive impairment, could not provide informed consent, or were asymptomatic were excluded from this study.

The standardized validated Malay language “Delayed Presentation” questionnaire [4], which was developed on the basis of expert discussions, were distributed among the participants. The questionnaire was pretested for face and content validity and reliability, which were satisfactory (Cronbach’s Alpha: 0.63–0.92). Completion of the questionnaire was facilitated by our medical officers in the BestARi clinic to minimize interviewer bias. The participants were given adequate time to read and answer the questionnaire with minimal contact with our researcher.

**Fig. 1:**
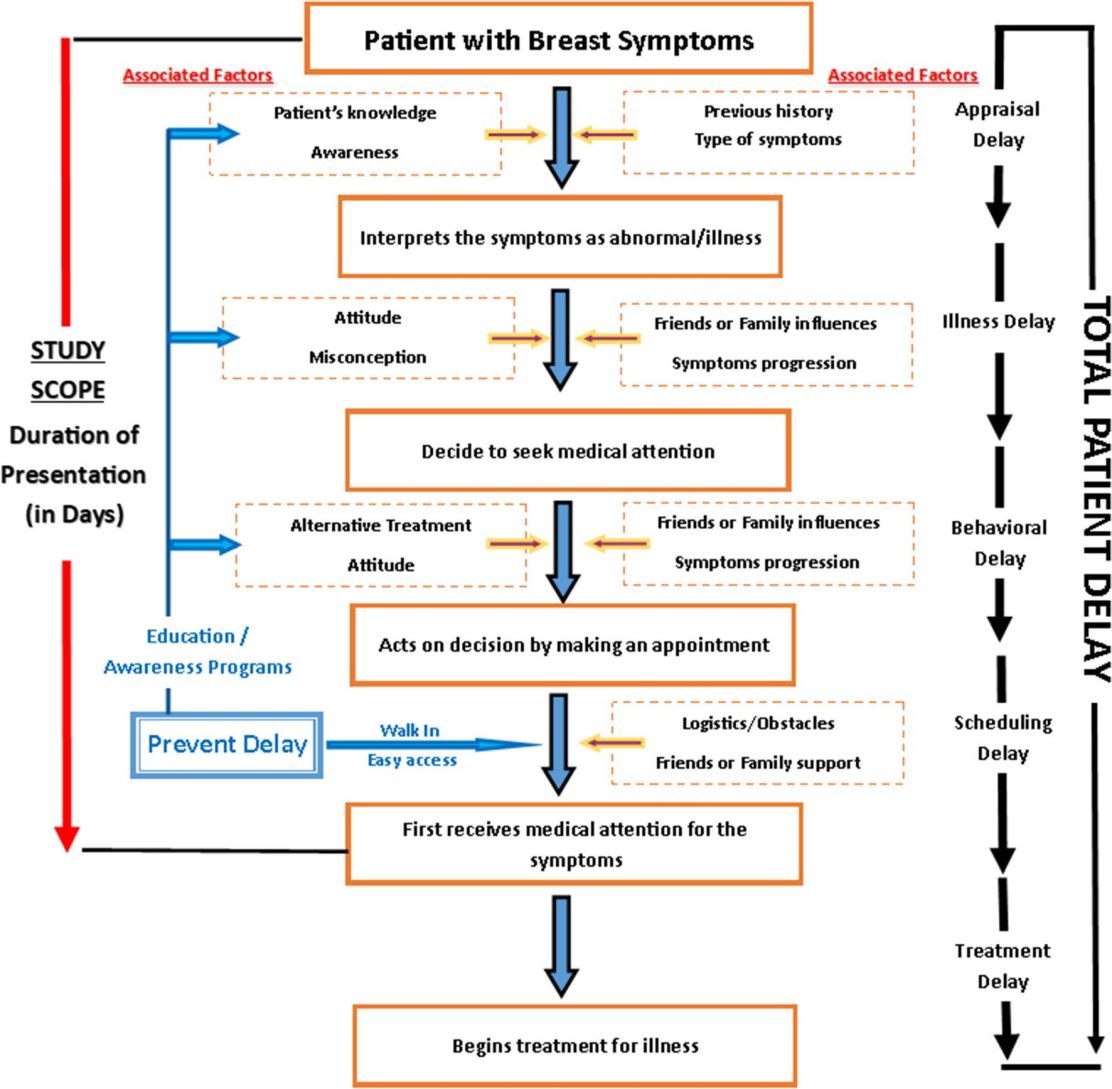
Study framework integrated with Total Patient Delay by Andersen and Cacioppo [7]

To reduce recall bias, we only recruited new cases or patients visiting for the first time to our BestARi clinic. Duration of days from the first day the patient self-discovered breast symptoms until the day the patient presented to any health facilities to seek evaluation were calculated. The questionnaire included items on sociodemographics, family history, medical and obstetric history, dates of all chronological events (first recognition of symptoms, first complaint to another person, and first doctor consultation), main reason or influences on seeking care, and use of alternative therapy. There were questions on the interpretation of symptoms, reasons for perceiving the symptom as not dangerous, attitude toward the health care workers or doctors, and the obstacles to health care services. The theoretical framework is illustrated in the diagram below (Fig. 1).

### Operational definition

The breast symptoms included were breast lumps, breast dimpling, Peau de orange, breast infection, nipple discharge, nipple indrawn, change in the shape of the breast, breast pain, breast ulcers, axillary lump, breast rashes, or breast itchiness [5, 6, 8, 9].

Presentation time was defined as the duration in days from the first day the patient self-discovered concerning breast symptoms until the day the patient presented to any health care facilities to seek evaluation [5].

Delayed presentation time was defined as a presentation time > 3 months (90 days) [6, 10, 11] before the first visit to the health clinic; these women were categorized as the delayed presentation group.

A family history of breast cancer was defined as having a first-degree or second-degree relative who had breast cancer. Alternative therapy was defined as any therapy using methods, health care systems, practices, and products not included in conventional modern medicine [12].

### Statistical analyses

Data were analyzed using SPSS for Windows (version 26, SPSS Inc., Chicago, IL, USA). Descriptive statistics was used to summarize the sociodemographics of patients. Continuous data were summarized as mean (standard deviation [SD]) or median depending upon the normality of distribution, whereas categorical data were presented as frequency (percentage [%]).

The presentation time was divided into a binary outcome of delay and nondelay using a 3-month cutoff point. Crosstabs of the associated factors with the delay presentation time were analyzed using chi-square test. Then, multiple logistic regression was used to identify the factors associated with presentation delay. The outcome of the analysis was binary: delay and nondelay with the cutoff presentation time of 3 months. A stepwise backward selection procedure was used when selecting significant variables for the model. The interaction terms and multicollinearity problem of the final model were checked. The final model was tested for fitness using the Hosmer–Lemeshow goodness of fit test.

Results were presented as the crude and adjusted odd ratios (OR), 95% confidence interval (CI), and *p* value. Statistical significance was set at *p* < 0.05.

## Results

### Sociodemographics

One hundred and eleven respondents were recruited into the study during the study period; however, five of them had incomplete data and were excluded. One hundred and six respondents were included in the final analysis. The respondents’ mean age was 33.99 (SD: 11.21) years. Forty-four (41.5%) respondents had education up to secondary school, and 69.8% were married. Majorities were either housewives (35.8%) or working as government servants (31.1%) (Table 1).

**Table 1:** Sociodemographic characteristics of the respondents

Social demography	Frequency (%) N = 106	Mean (SD)
Age (years)		33.99 (11.21)
< 29	41 (38.7)	
30 to 39	37 (34.9)	
40 to 49	16 (15.1)	
50 to 59	11 (10.4)	
≥ 70	1 (0.9)	
Ethnicity		
Malay	104 (98.1)	
Chinese	2 (1.9)	
Education level		
No formal education	1 (0.9)	
Primary school	0 (0)	
Secondary school	44 (41.5)	
Diploma	24 (22.6)	
Degree	37 (34.9)	
Occupation		
Housewife	38 (35.8)	
Government servant	33 (31.1)	
Private sector	11 (10.4)	
Self-employed	6 (5.7)	
Unemployed	18 (17.0)	
Marital status		
Not married	28 (26.0)	
Married	74 (69.8)	
Divorced	2 (1.9)	
Widowed	2 (1.9)	

### Patient history

In our study, 18.9% of the respondents had a family history of breast cancer, and 8.5% had a previous history of breast symptoms. Most of the respondents (79.2%) had spoken to another individual before attending the clinic, especially their husband (44.0%).

### Symptoms

The most common presentation symptom was a breast lump (75.5%), followed by pain (15.1%), nipple discharge (5.7%), and skin changes (0.9%). The main reason they sought care was that the lump was getting bigger (28.3%) or was associated with new symptoms (16.0%) (Table 2).

**Table 2:** Respondents’ symptoms

Symptoms	Frequency (%) N = 106
Symptoms during presentation	
Lump	80 (75.5)
Pain	16 (15.1)
Discharge	6 (5.7)
Skin changes	1 (0.9)
Others	3 (2.8)
Main reason for seeking care	
Lump bigger	30 (28.3)
Change in breast shape	8 (7.5)
Wound bigger	1 (0.9)
New symptoms	17 (16.0)
Loss of weight	1 (0.9)
Loss of appetite	1 (0.9)
News of breast cancer in media	15 (14.2)
Read about breast cancer	2 (1.9)
Family history of breast cancer	3 (2.8)
Advised by a friend	2 (1.9)
Advised by husband	3 (2.8)
Advised by family members	6 (5.7)
Others	17 (16.0)

### Presentation time to the health care

Most of the respondents visited the local government clinic (35.8%) for breast symptoms. Only 28.3% came to the BestARi clinic directly, and 24.5% went to a private clinic first. The decision to seek treatment was made by themselves (86.8%), and 88.7% decided immediately. Only 10.4% of the respondents sought alternative treatment. The mean presentation time was 98.91 (SD 323.7) days. The presentation time ranged from 1 to 3013 days, and 61.3% of the respondents presented to a clinic within 1 month. Only 19.8% of the respondents had delayed presentation > 3 months (Table 3).

**Table 3:** Respondents first seeking care for breast symptoms

First-time seeking care for symptoms	Frequency (%)
Type of health facility	
Private clinic	26 (24.5)
Local government clinic	38 (35.8)
District hospital	6 (5.7)
Government hospital	6 (5.7)
BESTARI	30 (28.3)
Sought care immediately	
Yes	94 (88.7)
No	12 (11.3)
Person who made the decision to seek care	
Self	92 (86.8)
Husband	2 (1.9)
Family member	12 (11.3)
Alternative treatment	
No	95 (89.6)
Yes	11 (10.4)
Presentation time	
0–1 month	65 (61.3)
> 1–3 months	20 (18.9)
> 3–6 months	9 (8.5)
> 6–12 months	6 (5.7)
> 12 months	6 (5.7)
Delayed presentation > 3 months	
Yes	21 (19.8)
No	85 (80.2)

### Associations of history, and symptoms with delay presentation time

Among the respondents with delayed presentation (n = 21), 23.8% had a family history of breast cancer, 9.5% had a previous history of concerning breast symptoms, 90.5% had a breast lump as the first symptom, and 19.0% sought alternative treatment. However, none of these factors was statistically significant (Table 4).

**Table 4:** Associations of previous history and symptoms with delayed presentation time

	Frequency (%)		*p *value	Crude OR (95% CI)	*p* value^c^
	No delay N = 85	Delay N = 21			
Family history of breast cancer					
No	70 (82.4)	16 (76.2)	0.539^b^	1.46 (0.46, 4.60)	0.520
Yes	15 (17.6)	5 (23.8)			
Previous history of breast symptoms					
No	78 (91.8)	19 (90.5)	> 0.95^b^	1.17 (0.23, 6.10)	0.850
Yes	7 (8.2)	2 (9.5)			
Lump as the first symptom					
No	24 (28.2)	2 (9.5)	0.074^a^	3.74 (0.81, 17.29)	0.092
Yes	61 (71.8)	19 (90.5)			
Alternative treatment					
No	78 (91.8)	17 (81.0)	0.222^b^	2.62 (0.69, 9.97)	0.157
Yes	7 (8.2)	4 (19.0)			

^a^Pearson’s Chi-Square Test

^b^Fisher’s Exact Test

^c^Simple Logistic Regression

### Interpretation of the symptoms and delayed presentation time

Among the respondents with delayed presentation, more than half thought the symptoms would resolve spontaneously (52.4%), were not dangerous (61.9%), or thought that cancer was not a possibility (61.9%). The interpretation of the symptom as not dangerous was a significant factor associated with presentation time (*p* = 0.044) (Table 5). The delay in presentation was because the respondent thought that the symptom was not dangerous because it was not painful (47.6%), because there were no other associated symptoms (61.9%), and because they felt that they were well (66.7%). However, there were no significant differences between these percentages (Table 6).

**Table 5:** Interpretation of the symptoms and presentation delay

Interpretation of the symptoms	Frequency (%)		*p *value	Crude OR (95% CI)	*p* value^c^
	No delay N = 85	Delay N = 21			
Resolved spontaneously					
No	42 (49.4)	10 (47.6)	0.883^a^	1.07 (0.41, 2.80)	0.883
Yes	43 (50.6)	11 (52.4)			
Not dangerous					
No	53 (62.4)	8 (38.1)	0.044^a^	2.69 (1.01, 7.20)	0.049
Yes	32 (37.6)	13 (61.9)			
No possibility of cancer					
No	51 (60.0)	8 (38.1)	0.070^a^	2.44 (0.91, 6.51)	0.075
Yes	34 (40.0)	13 (61.9)			
It is cancer					
No	69 (81.2)	18 (85.7)	0.759^b^	0.72 (0.19, 2.74)	0.629
Yes	16 (18.8)	3 (14.3)			
Breast engorgement					
No	56 (65.9)	14 (66.7)	0.946^a^	0.97 (0.35, 2.66)	0.946
Yes	29 (34.1)	7 (33.3)			
Infection					
No	69 (81.2)	19 (90.5)	0.517^b^	0.45(0.10, 2.15)	0.320
Yes	16 (18.8)	2 (9.5)			
Menstrual disturbance					
No	61 (71.8)	15 (71.4)	0.976^a^	1.02(0.35, 2.93)	0.976
Yes	24 (28.2)	6 (28.6)			
Muscle tension					
No	61 (71.8)	14 (66.7)	0.646^b^	1.27(0.46, 3.53)	0.646
Yes	24(28.2)	7 (33.3)			

^a^Pearson’s Chi-Square Test

^b^Fisher’s Exact Test

^c^Simple Logistic Regression

**Table 6:** Interpretation as not dangerous and presentation delay

Thought the symptom was not dangerous because	Frequency (%)		*p *value	Crude OR (95% CI)	*p* value^c^
	No delay N = 85	Delay N = 21			
It was not painful					
No	44 (51.8)	11 (52.4)	0.960^a^	0.98 (0.38, 2.54)	0.960
Yes	41 (48.2)	10 (47.6)			
No other associated symptoms					
No	42 (49.4)	8 (38.1)	0.352^a^	1.59 (0.60, 4.22)	0.355
Yes	43 (50.6)	13 (61.9)			
Feeling well					
No	28 (32.9)	7 (33.3)	0.973^a^	0.98 (0.36, 2.71)	0.973
Yes	57 (67.1)	14 (66.7)			

^a^Pearson’s Chi-Square Test

^b^Fisher’s Exact Test

^c^Simple Logistic Regression

### Attitudes toward health care providers

Overall, the respondents had a good attitude toward their health care providers. Among the respondents with delayed presentation, 38.1% were shy about having a breast examination, and 19.8% were concerned that most doctors in a health clinic were males, but this did not contribute to the delay in presentation. According to the respondent, their husbands were supportive, and 99.1% allowed them to undergo breast examination. The respondents also trusted modern treatment (98.1%) and did not think that alternative treatment was more effective (97.2%) (Table 7).

**Table 7:** Attitude toward health care provider

Attitude toward health care provider	Frequency (%)		*p *value	Crude OR (95% CI)	*p* value^c^
	No delay N = 85	Delay N = 21			
Most doctors are male					
No	66 (77.6)	19(90.5)	0.235^b^	0.37 (0.08, 1.71)	0.201
Yes	19 (22.4)	2 (9.5)			
Afraid the doctor will scold					
No	78 (91.8)	19 (90.5)	> 0.95^b^	1.17 (0.23, 6.11)	0.850
Yes	7 (8.2)	2 (9.5)			
The doctor was not friendly					
No	81 (95.3)	21 (100)	0.582^b^	–	–
Yes	4 (4.7)	0 (0)			
Shy in breast examination					
No	60 (70.6)	13 (61.9)	0.442^a^	1.48 (0.55, 4.00)	0.443
Yes	25 (29.4)	8 (38.1)			
Husband did not allow breast examination					
No	84 (98.8)	21 (100)	> 0.95^b^	–	–
Yes	1 (1.2)	0 (0.0)			
Did not trust modern treatment					
No	83 (97.6)	21 (100)	> 0.95^b^	–	–
Yes	2 (2.4)	0 (0)			
Believed that alternative (traditional) treatment is more effective in the treatment of breast illness					
No	82 (96.5)	21 (100)	> 0.95^b^	–	–
Yes	3 (3.5)	0 (0)			

^a^Pearson’s Chi-Square Test

^b^Fisher’s Exact Test

^c^Simple Logistic Regression

### Obstacles to obtaining care

In our study, 23.6% of the respondents claimed they had no time because they were always too busy with their work. Sixteen percent of the respondents complained of the long clinic waiting time, which caused difficulties in scheduling to see a doctor, 11.3% had a financial constraint, and 7.5% had many family problems. There were not many logistical issues among the respondents as only 4.7% said they did not know the clinic’s location, only 6.6% said the location was too far away from their home, and only 4.7% had no transport to the clinic. None of the obstacles was a statistically significant cause of the delay in presentation (Table 8).

**Table 8:** Obstacles to getting earlier care

Obstacle in getting care	Frequency (%)		*p *value	Crude OR (95% CI)	*p* value^c^
	No delay N = 85	Delay N = 21			
Did not know the location of clinic/hospital					
No	82 (96.5)	19 (90.5)	0.257^b^	2.88 (0.45, 18.44)	0.296
Yes	3 (3.5)	2 (9.5)			
Clinic/hospital far away from home					
No	81 (95.3)	18 (85.7)	0.138^b^	3.38 (0.69, 16.41)	0.132
Yes	4 (4.7)	3 (14.3)			
No transport					
No	81 (95.3)	20 (95.2)	> 0.95^b^	1.01 (0.11, 9.56)	0.991
Yes	4 (4.7)	1 (4.8)			
Long clinic waiting time					
No	72 (84.7)	17 (81.0)	0.741^b^	1.30 (0.38, 4.50)	0.675
Yes	13 (15.3)	4 (19.0)			
No time because too busy with work					
No	63 (74.1)	18 (85.7)	0.391^b^	0.48 (0.13, 1.78)	0.270
Yes	22 (25.9)	3 (14.3)			
Had many family problems					
No	79 (92.9)	19 (90.5)	0.656^b^	1.39 (0.26, 7.41)	0.703
Yes	6 (7.1)	2 (9.5)			
Financial constraint for the treatment and other cost					
No	76 (89.4)	18 (85.7)	0.701^b^	1.41 (0.35, 5.73)	0.633
Yes	9 (10.6)	3 (14.3)			

^a^Pearson’s Chi-Square Test

^b^Fisher’s Exact Test

^c^Simple Logistic Regression

### Factors associated with delayed presentation time

Variables with *p* values < 0.25 in the simple linear regression were included as variables in the multiple logistic regression modeling. The variables included were as follows: age > 40 years, breast lump as the first symptom, alternative treatment, interpretation of not dangerous, interpretation of no possibility for it to be cancer, concern that most doctors were male, not wanting to burden the doctor with a small matter, and clinic/hospital located too far away from home (Table 9).

**Table 9:** Simple logistic regression of potential factors associated with delayed presentation

Variable	Crude OR (95% CI)	*p *value
Age > 40 years	0.40 (0.11, 1.48)	0.170
Breast lump as the first symptom	3.74 (0.81, 17.29)	0.092
Alternative treatment	2.62 (0.69, 9.97)	0.157
Interpretation: not dangerous	2.69 (1.01, 7.20)	0.049
Interpretation: no possibility for it to be cancer	2.44 (0.91, 6.51)	0.075
Attitude: concern that most doctors are male	0.37 (0.08, 1.71)	0.201
Attitude: Did not want to burden the doctor with small matters	2.34 (0.72, 7.79)	0.165
Clinic/hospital too far away from home	3.38 (0.69, 16.41)	0.132

Multiple logistic regression analysis was performed (Table 10), and the only factor that was significantly associated with delayed presentation was the interpretation of the symptom as not being dangerous [adjusted OR (95% CI) 3.051 (1.111, 8.378); *p* = 0.030]. The results of the Hosmer–Lemeshow goodness of fit test showed that the selected model was a good fit.

**Table 10:** Multiple logistic regression modeling for the associated factors of delayed presentation of breast complaints

Factors causing delayed presentation	Adjusted OR (95% CI)	*p *value
Breast lump as the first symptom	4.39 (0.92, 20.92)	0.063
Interpretation: Not dangerous	3.05 (1.11, 8.38)	0.030

Backward LR method was applied

No interaction

Hosmer–Lemeshow test, *p* = 0.658

Classification table: 80.2% correctly classified

Area under the receiver operating characteristic curve: 67.7% (95% CI 0.56, 0.80)

## Discussion

Delay in seeking medical attention can be divided into five stages: appraisal delay, illness delay, behavioral delay, scheduling delay, and treatment delay [7]. The combination of these five stages is known as total patient delay, and the appraisal delay is the major stage, comprising 60% of the total delay [7].

Appraisal delay is the patient’s interpretation of her bodily symptoms as an illness or labeling it as serious symptoms [7, 9]. Illness delay is the number of days elapsing from the time an individual interpreted that her symptoms were concerning to the day she decided to seek medical attention, and behavioral delay is the time from her decision to the time she acted on the decision [7].

The first three stages, which are the appraisal, illness, and behavioral delay, are the patient-related delays. These stages comprised various factors such as the patient’s sociodemographics, previous history, interpretation of the breast symptoms, types of symptoms, knowledge, attitudes, and practices toward breast symptoms, which were analyzed in most previous studies [4, 6, 8, 9, 11, 13–15]. Lower educational level [16] and lack of knowledge [17] were associated with delayed presentation of breast cancer.

Most of the previous studies on delayed presentation were conducted only on women with diagnosed breast cancer. However, we included all women with any general breast symptoms in our study. We aimed to investigate the presentation time as any delay could impede the initial clinical assessment, subsequent investigation, and breast cancer detection. Women with any breast symptoms should present early to the clinic for proper assessment by the professional health care provider to determine whether the symptom is benign or malignant. There are possibilities which they might misinterpret malignant symptoms as benign thus leading to a delay in the diagnosis of breast cancer. Thus, it is important to seek medical attention early to plan the clinical approach.

The results of our study were compared with the previous studies whose population were women with breast cancer. In our study, the respondents’ mean age was 33.99 years, which was younger than the mean age of 47.9 years in a previous study [4]. This younger mean age could explain why the respondents in our study presented earlier to the clinic than older patients since younger patients probably were better educated and more aware of breast cancer risks. The predominantly young demographic, 73.6% were under 40 years of age, despite having a lower risk of breast cancer, they still presented earlier, better health-seeking behaviour compared to older cohort with a higher risk of developing breast cancer.

Our study found that 61.3% of the respondents presented within 1 month, and only 19.8% of the respondents had delayed presentation of > 3 months. This percentage was much lower than the percentage in a previous local study in Malaysia. This finding indicated the improving breast cancer awareness among the community and easily accessible health care service.

Our study was prospectively designed to avoid recall bias unlike most previous studies, which involved retrospective collection of data from medical records [4, 8, 9, 11, 15, 18, 19].

Presentation delay was operationally defined as the time elapsed between symptom self-discovery and the first presentation to a medical provider to seek evaluation [8]. A local multicenter study published in 2011 concluded that 43.4% of the patients with breast cancer had delayed presentation times [4].

A delay in presentation for breast cancer examination of > 3 months was previously found to be associated with 10% lower survival rates [9]. Another study estimated that 20% to 30% of women waited ≥ 3 months before seeking medical help with breast symptoms [11]. The delayed presentation group in our study had similar perceptions that their symptoms were not dangerous. This finding is consistent with the percentage of patients who misinterpreted their symptoms as less serious than cancer, which led to delayed presentation in a study with a Western population [5, 6].

Women that delayed > 3 months were less likely to have a breast lump and had a family member previously diagnosed with breast cancer [6]. We would expect that the respondents with a family history of breast cancer or a previous history of breast illness would present early to the clinic. Instead, we found that five (4.7%) respondents in our study with a family history of breast cancer had delayed presentations.

In our study, the most common symptom of our respondents was a breast lump (75.5%). The main reason for seeking care was that the lump was getting bigger. This finding suggests that there might be women with other breast symptoms who also do not seek medical attention. Women should understand that breast cancer symptoms are not limited to lumps and that there can be other symptoms, so early assessment is important for earlier diagnosis [9]. In a previous study, patients with breast symptoms other than a breast lump were likely to delay in presenting to a clinic [20]. However, in our study, we found the opposite. Our comparison of breast lumps to other breast symptoms showed that patients with breast lumps had a higher likelihood of delayed presentation (adjusted OR 4.39, 95% CI 0.92, 20.92; *p* = 0.063).

Most of the respondents informed their family members or friends about their symptoms prior to visiting a clinic. The husband can play an essential role in the decision to seek an examination when they first learn about their wife’s symptoms by encouraging them to present to a clinic as soon as possible. Nevertheless, the final decision to seek treatment was usually made by the women themselves.

Use of alternative medicine has previously been found to be associated with delayed presentation [4, 21]. In our study, only 10.4% of the respondents sought alternative treatments. A previous study in UMMC, Malaysia, found that the percentage of alternative medicine usage was 34.8% among newly diagnosed patients with breast cancer [12]. However, in our study, we included both benign and malignant conditions. The previous study cited above also noted that most (73.1%) of the respondents did not disclose their alternative medicine use to their doctor [12]. Our study population had a lower percentage of respondents who sought alternative treatment, which could be because they were a younger cohort that may have been less influenced by alternative health beliefs than an older cohort. However, we could only postulate this we did not explore this topic in our questionnaire. Our BestARi clinic is a friendly service that provides walk-in consultations, examinations, and treatments without a prior appointment for new cases. This “one-stop center” service was the preferred type of facility among the respondents.

Our study found that an interpretation of symptoms as harmless was associated with presentation delay (adjusted OR 3.051, 95% CI 1.111, 8.378). Thus, our future breast awareness campaign should highlight that any breast symptoms have the potential to be associated with a malignant breast cancer tumor. Our respondents had a good attitude toward doctors. A few respondents were concerned about the doctors being male, but this did not contribute to their delay in seeking treatment. The highest numbers (23.8%) of respondents in the delayed group did not want to burden the doctor with small matters. This finding could be due to their earlier interpretation of the symptom as not dangerous, thus not requiring medical attention. Many of the respondents felt shy in breast exanimation; therefore, it is crucial for health care facilities to maintain an environment that puts patients at ease and comfort. It is also essential for their husbands to support the breast examination and treatments, as many women first inform their husbands about their breast symptoms before visiting the clinic.

## Conclusions

Our study found a lower percentage of delayed presentation by women with breast symptoms than found in the previous studies on women with breast cancer in Malaysia. This finding reflects the improved breast cancer awareness among our community and our commitment to providing easily accessible health care services. The only factor significantly associated with delayed presentation was the perception of a symptom as being harmless. Future awareness campaigns should highlight that lumps in breasts are highly associated with malignant tumors, which should reduce the percentage of individuals who delay seeking examinations in the near future.

## Supplementary Information

**Additional file 1**. Supplementary Data.

## Data Availability

The datasets used and analyzed during the current study is available in the supplementary file (Additional file 1).

## Abbreviation

BestARi: Breast Cancer Awareness & Research Unit

## References

[1] Ferlay J, Soerjomataram I, Dikshit R, Eser S, Mathers C, Rebelo M, Parkin DM, Forman D, Bray F (2015). Cancer incidence and mortality worldwide: sources, methods and major patterns in GLOBOCAN 2012. Int J Cancer.

[2] Winters S, Martin C, Murphy D, Shokar NK (2017). Breast cancer epidemiology, prevention, and screening. Progress in molecular biology and translational science.

[3] Azizah AM, Hashimah B, Nirmal K, Siti Zubaidah AR, Puteri NA, Nabihah A et al. Malaysia Nationl Cancer Registry Report (MNCR) 2012-2016. 2019. p. 1-116.

[4] Norsa’adah B, Rampal KG, Rahmah MA, Naing NN, Biswal BM (2011). Diagnosis delay of breast cancer and its associated factors in Malaysian women. BMC Cancer.

[5] Nosarti C, Crayford T, Roberts JV, Elias E, McKenzie K, David AS (2000). Delay in presentation of symptomatic referrals to a breast clinic: patient and system factors. Br J Cancer.

[6] Meechan G, Collins J, Petrie KJ (2003). The relationship of symptoms and psychological factors to delay in seeking medical care for breast symptoms. Prev Med.

[7] Andersen BL, Cacioppo JT, Roberts DC (1995). Delay in seeking a cancer diagnosis: delay stages and psychophysiological comparison processes. Br J Soc Psychol.

[8] Lim JNW, Potrata B, Simonella L, Ng CWQ, Aw TC, Dahlui M, Hartman M, Mazlan R, Taib NA (2015). Barriers to early presentation of self-discovered breast cancer in Singapore and Malaysia: a qualitative multicentre study. BMJ Open.

[9] Taib NA, Yip CH, Low WY (2011). Recognising symptoms of breast cancer as a reason for delayed presentation in Asian women-the psycho-socio-cultural model for breast symptom appraisal: opportunities for intervention. Asian Pacific J Cancer Prev.

[10] Abu-Helalah AM, Alshraideh AH, Al-Hanaqtah M, Da’Na M, Al-Omari A, Mubaidin R (2016). Delay in presentation, diagnosis, and treatment for breast cancer patients in Jordan. Breast J.

[11] Burgess C, Hunter MS, Ramirez AJ (2001). A qualitative study of delay among women reporting symptoms of breast cancer. Br J Gen Pract.

[12] Zulkipli AF, Islam T, Mohd Taib NA, Dahlui M, Bhoo-Pathy N, Al-Sadat N, Abdul Majid H, Hussain S (2018). Use of complementary and alternative medicine among newly diagnosed breast cancer patients in Malaysia: an early report from the MyBCC study. Integr Cancer Ther.

[13] Arndt V, Stürmer T, Stegmaier C, Ziegler H, Dhom G, Brenner H (2002). Patient delay and stage of diagnosis among breast cancer patients in Germany—a population based study. Br J Cancer.

[14] Galukande M, Mirembe F, Wabinga H (2014). Patient delay in accessing breast cancer care in a Sub Saharan African country: Uganda. Br J Med Med Res.

[15] Hisham AN, Yip CH (2004). Overview of breastcancer in Malaysian women: a problem with late diagnosis. Asian J Surg.

[16] Montazeri A, Ebrahimi M, Mehrdad N, Ansari M, Sajadian A (2003). Delayed presentation in breast cancer: a study in Iranian women. BMC Womens Health.

[17] Baig M, Sohail I, Altaf HN, Altaf OS (2019). Factors influencing delayed presentation of breast cancer at a tertiary care hospital in Pakistan. Cancer Rep.

[18] Gould J, Fitzgerald B, Fergus K, Clemons M, Baig F (2010). Why women delay seeking assistance for locally advanced breast cancer. Can Oncol Nurs J.

[19] Tartter PI, Pace D, Frost M, Bernstein JL (1999). Delay in diagnosis of breast cancer. Ann Surg.

[20] Burgess CC, Ramirez AJ, Richards MA, Love SB (1998). Who and what influences delayed presentation in breast cancer?. Br J Cancer.

[21] Mastura N, Mujar M, Dahlui M, Emran NA, Hadi IA, Wai YY, Arulanantham S, Hooi CC, Mohd Taib NA. Complementary and alternative medicine ( CAM ) use and delays in presentation and diagnosis of breast cancer patients in public hospitals in Malaysia. PLoS ONE. 2017;1–12. 10.1371/journal.pone.0176394 PMC5407802 28448541

