# Supplementary material for: Factors associated with delay in seeking care for breast symptoms
Source: BMC Womens Health. 2022 Jul 27;22:316. doi: 10.1186/s12905-022-01898-5 (PMC9331147; doi:10.1186/s12905-022-01898-5)
Supplement: Supplementary file 1 — Additional file 1. Supplementary Data. [file 12905_2022_1898_MOESM1_ESM.pdf]

Supplementary Data

Table 1 Sociodemographic of the respondents

ID	Age	Age below or above 40	Age Group	Race	Education Level	Occupation	Marital Status
1	43	40 and above	40 to 49	Malay	University	Government	Married
2	22	39 and below	29 and below	Malay	Diploma	Private	Not Married
3	38	39 and below	30 to 39	Malay	Secondary	Private	Married
4	22	39 and below	29 and below	Malay	University	None	Not Married
5	32	39 and below	30 to 39	Malay	Secondary	Housewife	Married
6	30	39 and below	30 to 39	Malay	Secondary	Housewife	Married
7	34	39 and below	30 to 39	Malay	Diploma	Government	Married
8	49	40 and above	40 to 49	Malay	Secondary	Government	Not Married
9	23	39 and below	29 and below	Malay	Diploma	Self-Employed	Not Married
10	30	39 and below	30 to 39	Malay	Secondary	Private	Married
11	28	39 and below	29 and below	Malay	Secondary	Housewife	Married
12	29	39 and below	29 and below	Malay	Secondary	Private	Married
13	19	39 and below	29 and below	Malay	Diploma	None	Not Married
14	32	39 and below	30 to 39	Malay	University	Private	Married
15	29	39 and below	29 and below	Malay	Diploma	Housewife	Married
16	51	40 and above	50 to 59	Malay	University	Government	Married
17	21	39 and below	29 and below	Malay	University	Government	Not Married
18	30	39 and below	30 to 39	Malay	University	Private	Not Married
19	30	39 and below	30 to 39	Malay	University	Government	Married
20	20	39 and below	29 and below	Malay	University	None	Not Married
21	36	39 and below	30 to 39	Malay	Diploma	Government	Married
22	26	39 and below	29 and below	Malay	Secondary	Housewife	Married
23	28	39 and below	29 and below	Malay	University	None	Married
24	30	39 and below	30 to 39	Malay	Diploma	Government	Married
25	46	40 and above	40 to 49	Malay	Secondary	Housewife	Married
26	37	39 and below	30 to 39	Malay	Secondary	Government	Married
27	36	39 and below	30 to 39	Malay	Secondary	Housewife	Married
28	40	40 and above	40 to 49	Malay	Secondary	Government	Married
29	26	39 and below	29 and below	Malay	Secondary	Housewife	Married
30	23	39 and below	29 and below	Malay	University	None	Not Married
31	45	40 and above	40 to 49	Malay	Secondary	Government	Married
33	37	39 and below	30 to 39	Malay	Secondary	Housewife	Married
34	22	39 and below	29 and below	Malay	Diploma	Private	Not Married
35	46	40 and above	40 to 49	Malay	Secondary	Housewife	Married
36	31	39 and below	30 to 39	Malay	Secondary	Housewife	Married
37	45	40 and above	40 to 49	Malay	University	Government	Married
38	23	39 and below	29 and below	Malay	University	Self-Employed	Not Married
39	18	39 and below	29 and below	Malay	Secondary	None	Not Married
40	29	39 and below	29 and below	Malay	University	Private	Not Married
41	29	39 and below	29 and below	Malay	University	Housewife	Married
43	23	39 and below	29 and below	Malay	University	None	Not Married
44	26	39 and below	29 and below	Malay	University	Housewife	Married
45	35	39 and below	30 to 39	Malay	Secondary	Housewife	Married
46	21	39 and below	29 and below	Malay	Diploma	Housewife	Divorce
47	48	40 and above	40 to 49	Malay	Secondary	Self-Employed	Married
48	31	39 and below	30 to 39	Malay	University	Government	Married
49	31	39 and below	30 to 39	Malay	Secondary	Housewife	Married
50	47	40 and above	40 to 49	Malay	University	Government	Married
51	30	39 and below	30 to 39	Malay	University	Self-Employed	Married
52	25	39 and below	29 and below	Malay	Diploma	Private	Not Married
53	22	39 and below	29 and below	Malay	Secondary	None	Married
54	35	39 and below	30 to 39	Malay	University	Government	Married
55	38	39 and below	30 to 39	Malay	Diploma	Government	Married
56	26	39 and below	29 and below	Malay	Secondary	Housewife	Married
57	59	40 and above	50 to 59	Malay	Secondary	Housewife	Married
58	40	40 and above	40 to 49	Malay	Secondary	Self-Employed	Married
59	39	39 and below	30 to 39	Malay	University	Government	Married
60	42	40 and above	40 to 49	Malay	University	Government	Married
61	33	39 and below	30 to 39	Malay	Secondary	Private	Divorce
62	22	39 and below	29 and below	Malay	Secondary	Private	Not Married
63	20	39 and below	29 and below	Malay	Diploma	None	Not Married
64	26	39 and below	29 and below	Malay	Diploma	Housewife	Married
65	39	39 and below	30 to 39	Malay	University	Government	Married
66	44	40 and above	40 to 49	Malay	Secondary	Housewife	Married
67	52	40 and above	50 to 59	Chinese	Secondary	Housewife	Married
68	55	40 and above	50 to 59	Malay	No Formal Education	Housewife	Married
42	28	39 and below	29 and below	Malay	Diploma	Housewife	Married
69	18	39 and below	29 and below	Malay	Secondary	None	Not Married
71	20	39 and below	29 and below	Malay	Diploma	None	Not Married
72	52	40 and above	50 to 59	Malay	Secondary	Housewife	Married
83	34	39 and below	30 to 39	Malay	Diploma	Government	Not Married
84	34	39 and below	30 to 39	Malay	Secondary	Housewife	Married
85	35	39 and below	30 to 39	Malay	Secondary	Self-Employed	Married
86	34	39 and below	30 to 39	Malay	University	Government	Married
87	52	40 and above	50 to 59	Malay	University	Government	Widow
88	18	39 and below	29 and below	Malay	Secondary	None	Not Married
89	32	39 and below	30 to 39	Malay	Diploma	Housewife	Married
91	30	39 and below	30 to 39	Malay	University	Housewife	Married
92	29	39 and below	29 and below	Malay	Secondary	Housewife	Married
93	39	39 and below	30 to 39	Malay	Diploma	Government	Married
95	23	39 and below	29 and below	Malay	Secondary	Housewife	Married
96	74	40 and above	70 and above	Malay	Secondary	Housewife	Married
70	22	39 and below	29 and below	Malay	University	None	Not Married
73	50	40 and above	50 to 59	Malay	Secondary	Housewife	Married
74	30	39 and below	30 to 39	Malay	Secondary	Housewife	Married
75	22	39 and below	29 and below	Malay	University	None	Not Married
78	18	39 and below	29 and below	Malay	Diploma	None	Not Married
80	59	40 and above	50 to 59	Malay	Diploma	Government	Widow
81	38	39 and below	30 to 39	Malay	Secondary	Housewife	Married
82	20	39 and below	29 and below	Chinese	University	None	Not Married
97	34	39 and below	30 to 39	Malay	University	Housewife	Married
98	50	40 and above	50 to 59	Malay	Secondary	Government	Married
99	48	40 and above	40 to 49	Malay	University	Government	Married
100	49	40 and above	40 to 49	Malay	Diploma	Government	Married
101	52	40 and above	50 to 59	Malay	Secondary	Housewife	Married
102	52	40 and above	50 to 59	Malay	University	Government	Married
103	38	39 and below	30 to 39	Malay	Diploma	Government	Married
104	35	39 and below	30 to 39	Malay	University	Government	Married
105	42	40 and above	40 to 49	Malay	University	Government	Married
106	24	39 and below	29 and below	Malay	University	Government	Not Married
107	24	39 and below	29 and below	Malay	University	None	Not Married
108	26	39 and below	29 and below	Malay	University	None	Not Married
109	24	39 and below	29 and below	Malay	Diploma	Housewife	Married
110	49	40 and above	40 to 49	Malay	Secondary	Housewife	Married
111	34	39 and below	30 to 39	Malay	Secondary	Housewife	Married
94	37	39 and below	30 to 39	Malay	Diploma	Government	Married

Table 2 History of the respondents

ID	Family history of Breast Cancer	Breastfeeding	Menarche	Regular Menses	Menopause	OCP Usage	Hormonal Usage	History of any breast disease	Medical Comorbids	Smoker
1	No	Yes	12	Yes	No	No	No	No	No	No
2	No	No	14	Yes	No	No	No	No	No	No
3	Yes	Yes	15	No	No	No	No	No	No	No
4	No	No	15	Yes	No	No	No	No	No	No
5	Yes	Yes	13	Yes	No	No	No	No	No	No
6	Yes	Yes	9	Yes	No	No	No	No	No	No
7	No	Yes	12	Yes	No	No	No	No	No	No
8	Yes	No	16	No	No	No	No	No	No	No
9	Yes	No	13	Yes	No	No	No	No	No	No
10	No	No	12	Yes	No	No	No	No	No	No
11	Yes	No	14	No	No	No	No	No	No	No
12	No	Yes	11	No	No	Yes	No	No	No	No
13	No	No	13	Yes	No	No	No	No	No	No
14	Yes	Yes	13	No	No	No	No	No	No	No
15	No	Yes	13	Yes	No	No	No	Yes	No	No
16	No	Yes	12	No	No	No	No	No	Yes	No
17	No	No	13	Yes	No	No	No	No	No	No
18	No	No	13	No	No	No	No	No	No	No
19	No	Yes	13	Yes	No	No	No	No	No	No
20	No	No	11	Yes	No	No	No	No	No	No
21	No	Yes	12	Yes	No	Yes	No	No	No	No
22	No	Yes	12	Yes	No	Yes	No	No	No	No
23	No	No	12	Yes	No	No	No	Yes	No	No
24	Yes	No	17	Yes	No	No	No	No	No	No
25	No	Yes	13	Yes	No	Yes	No	No	No	No
26	No	Yes	13	Yes	No	Yes	No	No	No	No
27	Yes	Yes	12	Yes	No	No	No	No	No	No
28	No	Yes	12	No	No	Yes	No	No	Yes	No
29	No	Yes	12	Yes	No	No	No	No	No	No
30	No	No	12	Yes	No	No	No	No	No	No
31	No	Yes	14	Yes	No	No	No	No	No	No
33	No	Yes	12	Yes	No	No	No	No	No	No
34	No	No	9	No	No	No	No	No	No	No
35	No	No	12	Yes	No	No	No	No	No	No
36	No	Yes	13	Yes	No	No	No	No	No	No
37	No	Yes	12	Yes	No	Yes	No	No	No	No
38	Yes	No	12	No	No	No	No	No	No	No
39	No	No	11	No	No	No	No	No	No	No
40	Yes	No	12	Yes	No	No	No	No	No	No
41	No	No	12	No	No	No	No	Yes	No	No
43	No	No	14	Yes	No	No	No	No	No	No
44	No	Yes	11	Yes	No	No	No	No	No	No
45	No	Yes	10	No	No	Yes	No	No	Yes	No
46	No	No	12	Yes	No	No	No	No	No	No
47	No	Yes	12	Yes	Yes	No	No	No	No	No
48	No	Yes	14	No	No	No	No	Yes	No	No
49	No	Yes	14	No	No	Yes	No	No	No	No
50	No	Yes	13	Yes	No	Yes	No	No	No	No
51	No	No	13	Yes	No	No	No	No	No	No
52	No	No	12	Yes	No	No	No	No	No	No
53	Yes	Yes	13	No	No	No	No	No	No	No
54	No	Yes	14	Yes	No	No	No	Yes	No	No
55	No	Yes	14	No	No	Yes	No	No	No	No
56	No	No	14	Yes	No	No	No	No	No	No
57	Yes	Yes	12	Yes	Yes	Yes	No	No	Yes	No
58	No	Yes	14	Yes	No	Yes	No	No	No	No
59	No	Yes	12	No	No	No	No	No	No	No
60	No	Yes	12	Yes	No	No	No	No	No	No
61	No	Yes	12	Yes	No	Yes	No	No	Yes	No
62	No	No	11	No	No	No	No	No	No	No
63	Yes	No	12	Yes	No	No	No	No	No	No
64	No	Yes	12	Yes	No	No	No	No	No	No
65	Yes	Yes	12	No	No	No	No	No	No	No
66	No	Yes	14	Yes	No	No	No	No	No	No
67	No	No	12	No	Yes	No	No	No	No	No
68	No	Yes	16	Yes	No	Yes	No	No	No	No
42	No	Yes	11	Yes	No	No	No	No	No	No
69	No	No	13	Yes	No	No	No	Yes	No	No
71	No	No	12	Yes	No	No	No	No	No	No
72	No	Yes	13	Yes	No	No	No	No	Yes	No
83	No	No	12	No	No	No	No	No	No	No
84	No	Yes	14	Yes	No	No	No	No	No	No
85	No	Yes	16	Yes	No	No	No	No	No	No
86	Yes	Yes	11	No	No	No	No	No	No	No
87	No	Yes	12	Yes	No	No	No	No	Yes	No
88	No	No	14	No	No	No	No	No	No	No
89	No	Yes	16	Yes	No	No	No	No	No	No
91	No	Yes	12	No	No	No	No	No	No	No
92	No	Yes	14	Yes	No	Yes	No	No	No	No
93	No	Yes	15	Yes	No	Yes	No	Yes	No	No
95	No	Yes	14	Yes	No	No	No	No	No	No
96	No	Yes	15	Yes	Yes	No	No	No	No	No
70	No	No	14	No	No	Yes	No	No	No	No
73	No	Yes	13	Yes	No	Yes	No	No	Yes	No
74	No	Yes	13	No	No	Yes	No	No	Yes	No
75	Yes	No	13	Yes	No	No	No	No	No	No
78	Yes	No	13	Yes	No	No	No	No	No	No
80	No	Yes	12	No	Yes	No	No	No	No	No
81	No	Yes	15	Yes	No	No	No	No	No	No
82	No	No	14	Yes	No	No	No	No	No	No
97	No	No	12	Yes	No	No	No	No	No	No
98	No	Yes	14	Yes	No	Yes	No	No	No	No
99	No	Yes	14	No	No	Yes	No	No	No	No
100	Yes	Yes	12	Yes	No	Yes	No	No	Yes	No
101	No	Yes	14	No	No	No	No	No	No	No
102	No	Yes	10	Yes	No	No	No	No	No	No
103	No	Yes	14	Yes	No	Yes	No	No	No	No
104	No	Yes	15	Yes	No	Yes	No	No	No	No
105	No	No	12	Yes	No	No	No	No	No	No
106	Yes	No	12	Yes	No	No	No	No	No	No
107	No	No	13	Yes	No	No	No	Yes	No	No
108	No	No	13	No	No	No	No	No	No	No
109	No	Yes	13	Yes	No	No	No	No	No	No
110	No	Yes	12	Yes	No	No	No	No	Yes	No
111	No	No	13	Yes	No	No	No	Yes	Yes	No
94	No	Yes	14	Yes	No	No	No	No	No	No

Table 3 Respondents' symptoms

ID	First presented symptoms	Lump as first presentation?	Reason to seek treatment	Inform anyone	Inform to	Presentation Time(Day)	Presentation Delay	Presentation time range	First visit	Fisrt visit BESTARI ?	Immediate decision to see doctor	Whose decision to see doctor	Seek alternative treatment
1	Discharge	No	Others	No		1	No	0-1 months	Local Gov Clinic	No	Yes	Self	No
2	Lump	Yes	Others	Yes	Mother	20	No	0-1 months	Private Clinic	No	No	Family Member	No
3	Lump	Yes	Family history/friend with breast cancer	Yes	Husband	236	Yes	>6-12 months	BESTARI	Yes	No	Self	Yes
4	Lump	Yes	Advise by Friend	Yes	Friends	323	Yes	>6-12 months	BESTARI	Yes	No	Self	No
5	Lump	Yes	New symptoms	Yes	Husband	433	Yes	>12 months	Government Hospital	No	No	Self	Yes
6	Pain	No	Others	Yes	Husband	1	No	0-1 months	Local Gov Clinic	No	Yes	Self	No
7	Lump	Yes	New symptoms	Yes	Husband	2	No	0-1 months	BESTARI	Yes	Yes	Self	No
8	Lump	Yes	News of Breast Cancer in Media	Yes	Others	33	No	>1-3 months	BESTARI	Yes	Yes	Self	No
9	Lump	Yes	Change of breast shape	Yes	Mother	27	No	0-1 months	BESTARI	Yes	Yes	Self	No
10	Lump	Yes	Lump Bigger	Yes	Friends	3	No	0-1 months	Private Clinic	No	No	Self	Yes
11	Lump	Yes	Others	Yes	Mother	1	No	0-1 months	Government Hospital	No	No	Self	Yes
12	Lump	Yes	New symptoms	Yes	Husband	1	No	0-1 months	Local Gov Clinic	No	Yes	Self	No
13	Pain	No	Change of breast shape	Yes	Mother	6	No	0-1 months	BESTARI	Yes	Yes	Self	No
14	Lump	Yes	Lump Bigger	Yes	Mother	85	No	>1-3 months	District Hospital	No	Yes	Self	No
15	Lump	Yes	Lump Bigger	Yes	Mother	107	Yes	>3-6 months	Local Gov Clinic	No	Yes	Self	No
16	Pain	No	Loss of Appetite	Yes	Husband	50	No	>1-3 months	BESTARI	Yes	Yes	Self	No
17	Lump	Yes	News of Breast Cancer in Media	Yes	Mother	31	No	>1-3 months	BESTARI	Yes	Yes	Self	No
18	Lump	Yes	Others	No		365	Yes	>6-12 months	Private Clinic	No	No	Self	Yes
19	Lump	Yes	Lump Bigger	Yes	Husband	2	No	0-1 months	Local Gov Clinic	No	Yes	Self	No
20	Lump	Yes	News of Breast Cancer in Media	Yes	Sister	1	No	0-1 months	Private Clinic	No	Yes	Self	No
21	Discharge	No	Others	No		3	No	0-1 months	Government Hospital	No	No	Self	No
22	Discharge	No	Advise by Other Family members	Yes	Sister	6	No	0-1 months	District Hospital	No	Yes	Family Member	No
23	Lump	Yes	Lump Bigger	Yes	Husband	83	No	>1-3 months	BESTARI	Yes	Yes	Husband	No
24	Lump	Yes	Lump Bigger	Yes	Husband	75	No	>1-3 months	BESTARI	Yes	No	Self	No
25	Lump	Yes	Lump Bigger	Yes	Husband	1	No	0-1 months	Private Clinic	No	Yes	Self	No
26	Lump	Yes	New symptoms	Yes	Friends	54	No	>1-3 months	Local Gov Clinic	No	Yes	Self	No
27	Lump	Yes	Lump Bigger	No		44	No	>1-3 months	Private Clinic	No	Yes	Self	No
28	Lump	Yes	Lump Bigger	Yes	Husband	61	No	>1-3 months	Local Gov Clinic	No	Yes	Self	No
29	Lump	Yes	Lump Bigger	No		67	No	>1-3 months	Private Clinic	No	Yes	Self	No
30	Lump	Yes	New symptoms	Yes	Mother	9	No	0-1 months	Private Clinic	No	Yes	Self	No
31	Lump	Yes	Advise by Husband	Yes	Husband	5	No	0-1 months	Local Gov Clinic	No	Yes	Self	No
33	Lump	Yes	New symptoms	Yes	Husband	6	No	0-1 months	BESTARI	Yes	Yes	Self	No
34	Skin changes	No	Wound bigger	Yes	Others	4	No	0-1 months	Private Clinic	No	No	Family Member	No
35	Lump	Yes	Lump Bigger	No		8	No	0-1 months	BESTARI	Yes	Yes	Self	Yes
36	Pain	No	Reading about breast cancer in printed media	No		761	Yes	>12 months	BESTARI	Yes	Yes	Self	No
37	Lump	Yes	Lump Bigger	No		456	Yes	>12 months	Local Gov Clinic	No	Yes	Self	No
38	Lump	Yes	Lump Bigger	Yes	Mother	2	No	0-1 months	Local Gov Clinic	No	Yes	Self	Yes
39	Lump	Yes	Advise by Other Family members	Yes	Mother	1	No	0-1 months	Private Clinic	No	Yes	Self	No
40	Lump	Yes	Family history/friend with breast cancer	Yes	Others	1	No	0-1 months	Private Clinic	No	Yes	Self	No
41	Lump	Yes	Lump Bigger	Yes	Husband	1	No	0-1 months	Local Gov Clinic	No	Yes	Self	No
43	Lump	Yes	Lump Bigger	Yes	Mother	503	Yes	>12 months	Local Gov Clinic	No	Yes	Self	No
44	Lump	Yes	Lump Bigger	Yes	Husband	2	No	0-1 months	BESTARI	Yes	Yes	Self	No
45	Lump	Yes	Others	Yes	Others	41	No	>1-3 months	BESTARI	Yes	Yes	Self	No
46	Lump	Yes	Others	No		50	No	>1-3 months	BESTARI	Yes	Yes	Self	No
47	Pain	No	Change of breast shape	Yes	Husband	39	No	>1-3 months	Local Gov Clinic	No	Yes	Self	No
48	Lump	Yes	Lump Bigger	Yes	Husband	365	Yes	>6-12 months	Private Clinic	No	Yes	Self	No
49	Lump	Yes	Advise by Husband	Yes	Others	250	Yes	>6-12 months	Local Gov Clinic	No	Yes	Husband	Yes
50	Lump	Yes	Lump Bigger	Yes	Others	74	No	>1-3 months	Private Clinic	No	Yes	Self	No
51	Lump	Yes	News of Breast Cancer in Media	No		3013	Yes	>12 months	Health Clinic	No	Yes	Self	No
52	Lump	Yes	News of Breast Cancer in Media	Yes	Mother	88	No	>1-3 months	BESTARI	Yes	Yes	Self	No
53	Lump	Yes	New symptoms	No		15	No	0-1 months	BESTARI	Yes	Yes	Self	No
54	Lump	Yes	Advise by Husband	Yes	Husband	35	No	>1-3 months	Private Clinic	No	Yes	Self	No
55	Lump	Yes	New symptoms	Yes	Husband	92	Yes	>3-6 months	Local Gov Clinic	No	Yes	Self	No
56	Pain	No	News of Breast Cancer in Media	Yes	Mother	1	No	0-1 months	Local Gov Clinic	No	Yes	Family Member	No
57	Lump	Yes	Others	Yes	Husband	94	Yes	>3-6 months	Local Gov Clinic	No	Yes	Self	No
58	Pain	No	Reading about breast cancer in printed media	No		25	No	0-1 months	Local Gov Clinic	No	Yes	Self	No
59	Lump	Yes	News of Breast Cancer in Media	Yes	Mother	92	Yes	>3-6 months	Private Clinic	No	Yes	Self	No
60	Lump	Yes	Lump Bigger	Yes	Husband	29	No	0-1 months	BESTARI	Yes	Yes	Self	No
61	Pain	No	Advise by Other Family members	Yes	Others	13	No	0-1 months	Local Gov Clinic	No	Yes	Family Member	No
62	Lump	Yes	Lump Bigger	Yes	Mother	15	No	0-1 months	Private Clinic	No	Yes	Family Member	No
63	Discharge	No	Change of breast shape	Yes	Mother	99	Yes	>3-6 months	District Hospital	No	Yes	Family Member	No
64	Lump	Yes	Lump Bigger	Yes	Husband	14	No	0-1 months	District Hospital	No	Yes	Self	No
65	Pain	No	Others	Yes	Husband	1	No	0-1 months	Government Hospital	No	Yes	Self	No
66	Discharge	No	Advise by Other Family members	Yes	Sister	4	No	0-1 months	Local Gov Clinic	No	Yes	Self	Yes
67	Others	No	News of Breast Cancer in Media	Yes	Husband	1	No	0-1 months	Private Clinic	No	Yes	Self	No
68	Lump	Yes	Lump Bigger	No		5	No	0-1 months	Private Clinic	No	Yes	Self	No
42	Lump	Yes	Lump Bigger	Yes	Husband	109	Yes	>3-6 months	Private Clinic	No	Yes	Self	No
69	Pain	No	Loss of Weight	Yes	Mother	29	No	0-1 months	Local Gov Clinic	No	Yes	Self	No
71	Lump	Yes	Advise by Friend	Yes	Friends	265	Yes	>6-12 months	BESTARI	Yes	Yes	Self	No
72	Lump	Yes	Change of breast shape	Yes	Husband	115	Yes	>3-6 months	Local Gov Clinic	No	Yes	Self	No
83	Pain	No	Others	Yes	Others	7	No	0-1 months	Local Gov Clinic	No	Yes	Self	No
84	Pain	No	Advise by Other Family members	Yes	Sister	2	No	0-1 months	District Hospital	No	Yes	Self	No
85	Pain	No	News of Breast Cancer in Media	Yes	Mother	2	No	0-1 months	Government Hospital	No	Yes	Self	No
86	Lump	Yes	News of Breast Cancer in Media	Yes	Mother	31	No	>1-3 months	Local Gov Clinic	No	No	Self	No
87	Pain	No	New symptoms	Yes	Others	2	No	0-1 months	Local Gov Clinic	No	Yes	Self	No
88	Lump	Yes	Others	No		1	No	0-1 months	Private Clinic	No	Yes	Family Member	No
89	Lump	Yes	Others	Yes	Mother	2	No	0-1 months	Local Gov Clinic	No	Yes	Self	No
91	Lump	Yes	Lump Bigger	Yes	Husband	5	No	0-1 months	Private Clinic	No	Yes	Self	No
92	Lump	Yes	Others	Yes	Husband	1	No	0-1 months	Local Gov Clinic	No	Yes	Self	No
93	Lump	Yes	New symptoms	No		9	No	0-1 months	Local Gov Clinic	No	Yes	Self	No
95	Lump	Yes	Lump Bigger	Yes	Husband	3	No	0-1 months	District Hospital	No	Yes	Self	No
96	Lump	Yes	Change of breast shape	Yes	Others	10	No	0-1 months	Private Clinic	No	Yes	Self	No
70	Lump	Yes	Advise by Other Family members	Yes	Husband	30	No	0-1 months	Local Gov Clinic	No	Yes	Family Member	No
73	Lump	Yes	News of Breast Cancer in Media	No		18	No	0-1 months	BESTARI	Yes	Yes	Self	No
74	Pain	No	Change of breast shape	No		41	No	>1-3 months	Local Gov Clinic	No	Yes	Self	No
75	Lump	Yes	Lump Bigger	Yes	Mother	19	No	0-1 months	BESTARI	Yes	Yes	Self	No
78	Lump	Yes	Lump Bigger	Yes	Mother	99	Yes	>3-6 months	Private Clinic	No	Yes	Family Member	No
80	Discharge	No	New symptoms	Yes	Others	31	No	>1-3 months	BESTARI	Yes	Yes	Self	No
81	Lump	Yes	News of Breast Cancer in Media	No		6	No	0-1 months	BESTARI	Yes	Yes	Self	No
82	Lump	Yes	News of Breast Cancer in Media	Yes	Mother	37	No	>1-3 months	Local Gov Clinic	No	Yes	Family Member	No
97	Lump	Yes	Others	No		9	No	0-1 months	Local Gov Clinic	No	Yes	Self	No
98	Lump	Yes	Lump Bigger	Yes	Others	21	No	0-1 months	Private Clinic	No	Yes	Self	No
99	Lump	Yes	Others	No		14	No	0-1 months	Private Clinic	No	Yes	Self	No
100	Others	No	Family history/friend with breast cancer	Yes	Others	4	No	0-1 months	BESTARI	Yes	Yes	Self	No
101	Lump	Yes	Lump Bigger	Yes	Husband	4	No	0-1 months	Local Gov Clinic	No	Yes	Self	No
102	Pain	No	New symptoms	Yes	Sister	7	No	0-1 months	Local Gov Clinic	No	Yes	Self	No
103	Lump	Yes	New symptoms	Yes	Husband	141	Yes	>3-6 months	Local Gov Clinic	No	No	Family Member	No
104	Lump	Yes	Change of breast shape	Yes	Husband	4	No	0-1 months	BESTARI	Yes	Yes	Self	No
105	Lump	Yes	Lump Bigger	Yes	Husband	24	No	0-1 months	Private Clinic	No	Yes	Self	Yes
106	Others	No	New symptoms	No		1	No	0-1 months	BESTARI	Yes	Yes	Self	No
107	Lump	Yes	Others	Yes	Others	1	No	0-1 months	Government Hospital	No	Yes	Self	No
108	Lump	Yes	News of Breast Cancer in Media	No		1001	Yes	>12 months	BESTARI	Yes	Yes	Self	No
109	Lump	Yes	New symptoms	Yes	Husband	13	No	0-1 months	Local Gov Clinic	No	Yes	Self	No
110	Lump	Yes	News of Breast Cancer in Media	Yes	Others	7	No	0-1 months	BESTARI	Yes	Yes	Self	Yes
111	Lump	Yes	New symptoms	Yes	Husband	13	No	0-1 months	Local Gov Clinic	No	Yes	Self	No
94	Lump	Yes	New symptoms	Yes	Husband	10	No	0-1 months	BESTARI	Yes	Yes	Self	No

Table 4 Interpretation of symptoms

ID	Will spontaneous resolve	Non dangerous	No possibility of cancer	It is cancer	Breast engorgement	Infection	Mesntrual disturbance	Nerve entrapment	Muscle tense	Non painful	No associated symptoms	Feeling healthy
1	No	No	No	No	No	Yes	No	No	No	Yes	Yes	Yes
2	Yes	Yes	Yes	No	No	No	No	No	No	Yes	No	Yes
3	No	Yes	Yes	No	No	No	Yes	No	No	Yes	No	Yes
4	No	Yes	Yes	No	No	No	No	No	No	Yes	Yes	Yes
5	Yes	Yes	Yes	No	Yes	No	No	Yes	Yes	No	Yes	Yes
6	Yes	Yes	Yes	No	Yes	Yes	No	Yes	Yes	No	Yes	Yes
7	No	No	Yes	No	Yes	No	No	No	No	No	No	Yes
8	No	No	No	Yes	No	No	No	No	No	Yes	Yes	Yes
9	No	No	No	No	No	No	Yes	No	No	Yes	Yes	Yes
10	Yes	Yes	Yes	No	Yes	No	No	No	No	Yes	No	No
11	Yes	No	Yes	No	No	No	Yes	Yes	Yes	Yes	No	Yes
12	No	No	Yes	No	No	No	Yes	Yes	Yes	Yes	Yes	Yes
13	Yes	Yes	Yes	No	No	No	No	Yes	Yes	Yes	Yes	Yes
14	No	No	No	No	Yes	No	No	No	No	Yes	No	No
15	Yes	Yes	Yes	No	No	No	No	Yes	Yes	Yes	Yes	Yes
16	Yes	Yes	Yes	No	Yes	Yes	Yes	Yes	No	No	Yes	No
17	No	No	No	No	No	No	Yes	No	No	No	No	No
18	Yes	Yes	Yes	No	No	No	No	No	No	Yes	Yes	Yes
19	Yes	Yes	No	No	Yes	No	No	No	No	No	No	Yes
20	Yes	Yes	Yes	No	No	No	No	No	Yes	Yes	Yes	Yes
21	Yes	Yes	No	No	No	Yes	No	No	No	No	Yes	Yes
22	Yes	No	No	No	No	No	No	No	No	No	No	Yes
23	Yes	Yes	Yes	Yes	Yes	Yes	Yes	Yes	Yes	No	Yes	Yes
24	No	No	No	No	No	No	No	No	No	No	Yes	No
25	No	No	Yes	No	No	No	No	No	No	No	No	Yes
26	No	No	No	No	No	No	No	No	No	No	No	No
27	No	No	No	No	Yes	No	No	No	No	No	No	Yes
28	Yes	Yes	No	No	No	No	No	No	No	Yes	Yes	Yes
29	Yes	Yes	No	No	Yes	No	No	Yes	Yes	No	No	No
30	No	No	No	No	No	No	Yes	No	No	Yes	Yes	Yes
31	Yes	Yes	No	No	Yes	No	Yes	No	No	No	No	Yes
33	No	No	No	No	Yes	No	Yes	Yes	Yes	No	No	Yes
34	Yes	Yes	Yes	No	No	Yes	No	No	No	No	Yes	No
35	No	Yes	Yes	Yes	No	No	No	Yes	No	No	No	No
36	Yes	Yes	Yes	No	Yes	No	Yes	Yes	Yes	No	No	Yes
37	No	No	No	No	No	No	No	No	No	Yes	Yes	Yes
38	Yes	Yes	Yes	Yes	Yes	Yes	Yes	Yes	Yes	Yes	Yes	Yes
39	No	Yes	No	No	No	No	Yes	No	No	Yes	Yes	Yes
40	No	No	Yes	Yes	Yes	No	Yes	Yes	Yes	Yes	Yes	Yes
41	No	No	No	No	Yes	No	No	No	No	Yes	Yes	Yes
43	No	No	No	No	Yes	No	No	No	No	No	Yes	No
44	Yes	Yes	Yes	No	No	Yes	No	Yes	Yes	No	No	Yes
45	No	No	No	Yes	No	No	No	No	No	No	No	No
46	No	No	No	No	No	No	No	No	No	Yes	Yes	Yes
47	No	Yes	No	No	No	No	No	Yes	Yes	Yes	Yes	No
48	No	No	Yes	Yes	Yes	Yes	Yes	Yes	Yes	No	No	No
49	Yes	No	No	No	No	No	No	No	No	No	Yes	No
50	No	No	Yes	No	Yes	No	No	No	No	Yes	Yes	Yes
51	No	Yes	No	No	No	No	No	No	No	Yes	Yes	Yes
52	Yes	No	No	No	No	No	Yes	No	No	No	No	Yes
53	Yes	Yes	No	No	No	No	No	No	No	Yes	No	Yes
54	No	No	No	No	No	No	No	No	No	Yes	Yes	Yes
55	Yes	Yes	No	No	Yes	No	Yes	No	No	Yes	Yes	Yes
56	Yes	Yes	Yes	No	No	No	Yes	Yes	No	Yes	Yes	Yes
57	No	No	No	Yes	No	No	No	No	No	No	No	No
58	No	Yes	Yes	Yes	No	No	No	No	No	Yes	Yes	Yes
59	No	No	Yes	No	No	No	No	Yes	Yes	No	No	No
60	No	No	No	No	No	No	No	No	No	No	No	No
61	Yes	No	No	No	No	No	No	No	No	No	No	Yes
62	Yes	Yes	No	No	No	No	Yes	No	Yes	Yes	Yes	Yes
63	Yes	Yes	Yes	No	No	Yes	No	Yes	Yes	No	Yes	Yes
64	Yes	No	No	No	No	No	No	No	No	No	No	Yes
65	No	No	No	Yes	Yes	Yes	No	No	No	No	No	No
66	Yes	No	No	No	Yes	No	No	No	No	Yes	No	No
67	No	No	No	Yes	No	No	No	No	No	No	No	No
68	No	Yes	No	No	No	No	No	Yes	No	Yes	Yes	Yes
42	Yes	No	No	No	No	No	No	No	No	Yes	No	No
69	Yes	No	Yes	No	No	No	No	No	No	No	No	Yes
71	Yes	Yes	Yes	Yes	No	No	Yes	Yes	No	No	Yes	Yes
72	No	No	No	No	No	No	No	No	No	No	No	No
83	Yes	No	Yes	Yes	Yes	No	Yes	Yes	Yes	No	Yes	No
84	Yes	Yes	No	Yes	No	Yes	No	Yes	Yes	No	Yes	No
85	Yes	No	Yes	No	No	No	No	Yes	Yes	No	No	No
86	Yes	No	No	No	Yes	No	No	No	Yes	No	Yes	No
87	No	No	Yes	No	Yes	No	Yes	Yes	Yes	No	No	Yes
88	No	No	No	No	No	No	No	Yes	No	No	No	Yes
89	No	No	No	No	Yes	No	No	No	No	No	Yes	No
91	Yes	Yes	Yes	No	Yes	No	No	No	No	No	Yes	Yes
92	No	No	No	No	No	No	No	No	No	Yes	No	No
93	No	No	No	No	No	No	No	No	Yes	No	No	No
95	Yes	No	Yes	No	No	No	No	No	No	No	No	Yes
96	Yes	Yes	Yes	No	No	No	No	No	No	Yes	No	No
70	Yes	Yes	Yes	No	Yes	Yes	Yes	No	No	No	Yes	Yes
73	No	No	No	No	Yes	No	No	No	No	Yes	No	Yes
74	Yes	Yes	Yes	No	No	Yes	No	No	No	No	Yes	Yes
75	Yes	No	Yes	Yes	Yes	Yes	No	No	No	Yes	Yes	Yes
78	Yes	Yes	Yes	No	Yes	No	No	No	Yes	No	No	Yes
80	Yes	No	No	No	No	No	No	No	No	Yes	Yes	Yes
81	Yes	No	Yes	No	Yes	No	No	No	No	Yes	No	Yes
82	No	Yes	No	No	No	No	No	No	No	Yes	Yes	Yes
97	Yes	Yes	Yes	No	Yes	No	No	Yes	Yes	Yes	Yes	Yes
98	No	No	Yes	No	No	No	No	No	No	Yes	Yes	Yes
99	Yes	No	No	No	No	Yes	Yes	No	No	Yes	No	Yes
100	No	No	No	No	No	No	No	No	No	Yes	No	No
101	Yes	Yes	No	No	No	No	No	Yes	Yes	No	No	No
102	Yes	Yes	No	No	No	No	Yes	Yes	No	No	Yes	No
103	Yes	Yes	Yes	No	Yes	No	No	No	No	Yes	Yes	Yes
104	No	No	Yes	Yes	No	No	No	No	No	Yes	Yes	Yes
105	No	No	No	No	No	No	No	No	Yes	No	No	No
106	No	No	No	No	No	Yes	No	No	No	Yes	Yes	Yes
107	No	No	No	Yes	Yes	Yes	Yes	No	No	No	No	Yes
108	No	Yes	Yes	No	No	No	Yes	No	No	Yes	Yes	Yes
109	Yes	No	Yes	No	Yes	No	Yes	No	No	Yes	Yes	Yes
110	Yes	No	No	Yes	No	No	Yes	Yes	Yes	No	Yes	Yes
111	No	No	No	Yes	No	No	Yes	Yes	Yes	No	No	Yes
94	No	No	No	No	No	No	No	No	No	Yes	No	No

Table 5 Attitude toward health care provider

ID	Doctor mostly male	Afraid the doctor will scold	The doctor was not friendly	Do not know any doctor	No family doctor	Shy in breast examination	Husband did not allow breast examination	Did not trust modern treatment	Believed that alternative (traditional) treatment is more effective
1	Yes	No	No	No	No	No	No	No	No
2	Yes	No	No	No	No	Yes	No	No	No
3	No	No	No	No	No	No	No	No	No
4	No	No	No	Yes	Yes	Yes	No	No	No
5	No	Yes	No	No	No	No	No	No	No
6	Yes	No	Yes	No	No	Yes	No	No	No
7	No	No	No	Yes	Yes	No	No	No	No
8	No	No	No	No	No	No	No	No	No
9	No	No	No	No	No	No	No	No	No
10	No	No	No	Yes	Yes	Yes	No	No	No
11	Yes	No	No	Yes	Yes	Yes	No	No	Yes
12	No	No	No	No	No	No	No	No	No
13	No	No	No	No	No	Yes	No	No	No
14	No	No	No	No	No	Yes	No	No	No
15	No	No	No	No	No	No	No	No	No
16	Yes	Yes	No	Yes	Yes	No	No	No	No
17	No	No	No	No	No	No	No	No	No
18	No	No	No	No	No	Yes	No	No	No
19	Yes	No	Yes	Yes	Yes	No	No	No	No
20	No	No	No	No	Yes	Yes	No	No	No
21	No	No	No	Yes	Yes	Yes	No	No	No
22	No	No	No	No	No	Yes	No	No	Yes
23	No	No	No	Yes	Yes	No	No	No	No
24	No	No	No	No	No	Yes	No	No	No
25	No	No	No	No	No	Yes	No	No	No
26	No	No	No	No	No	No	No	No	No
27	No	No	No	No	No	No	No	No	No
28	Yes	No	No	No	No	No	No	No	No
29	No	No	No	No	No	No	No	No	No
30	No	No	No	No	No	No	No	No	No
31	No	No	No	Yes	Yes	No	No	No	No
33	No	No	No	No	No	No	No	No	No
34	No	No	No	Yes	Yes	No	No	No	No
35	No	No	No	Yes	Yes	No	No	Yes	Yes
36	No	No	No	Yes	Yes	Yes	No	No	No
37	No	No	No	No	No	No	No	No	No
38	Yes	Yes	No	No	No	Yes	No	No	No
39	No	No	No	No	No	No	No	No	No
40	Yes	No	No	No	No	No	No	No	No
41	No	No	No	No	No	No	No	No	No
43	No	No	No	No	No	Yes	No	No	No
44	No	No	No	No	No	No	No	No	No
45	No	No	No	Yes	No	No	No	No	No
46	No	No	No	No	No	No	No	No	No
47	No	No	No	No	No	No	No	Yes	No
48	No	No	No	No	No	No	No	No	No
49	No	No	No	No	No	Yes	No	No	No
50	No	No	No	Yes	Yes	Yes	Yes	No	No
51	No	No	No	Yes	No	Yes	No	No	No
52	No	No	No	No	No	No	No	No	No
53	No	No	No	No	No	No	No	No	No
54	Yes	No	No	No	No	No	No	No	No
55	No	No	No	No	No	No	No	No	No
56	No	No	No	No	Yes	No	No	No	No
57	No	No	No	No	No	No	No	No	No
58	No	No	No	No	No	No	No	No	No
59	No	No	No	No	No	No	No	No	No
60	No	No	No	No	No	No	No	No	No
61	No	No	No	No	No	Yes	No	No	No
62	Yes	Yes	No	No	No	No	No	No	No
63	No	No	No	No	No	No	No	No	No
64	No	No	No	No	No	No	No	No	No
65	No	No	No	No	No	No	No	No	No
66	Yes	No	No	No	No	Yes	No	No	No
67	Yes	No	No	No	No	Yes	No	No	No
68	No	No	No	No	No	No	No	No	No
42	No	No	No	No	No	No	No	No	No
69	No	Yes	No	Yes	Yes	Yes	No	No	No
71	No	Yes	No	No	Yes	Yes	No	No	No
72	No	No	No	No	No	No	No	No	No
83	No	No	No	No	No	No	No	No	No
84	No	No	No	No	No	No	No	No	No
85	Yes	No	No	Yes	Yes	No	No	No	No
86	No	No	No	No	No	No	No	No	No
87	Yes	No	No	Yes	Yes	Yes	No	No	No
88	Yes	No	No	No	No	No	No	No	No
89	No	No	No	No	No	No	No	No	No
91	Yes	No	No	Yes	Yes	Yes	No	No	No
92	No	No	No	No	No	No	No	No	No
93	No	No	No	No	No	No	No	No	No
95	No	No	No	No	Yes	No	No	No	No
96	No	Yes	No	No	No	Yes	No	No	No
70	Yes	Yes	No	Yes	No	Yes	No	No	No
73	No	No	No	No	No	No	No	No	No
74	No	No	No	Yes	No	No	No	No	No
75	No	No	No	No	No	No	No	No	No
78	Yes	No	No	No	No	Yes	No	No	No
80	No	No	No	Yes	Yes	Yes	No	No	No
81	No	No	No	Yes	No	Yes	No	No	No
82	No	No	No	No	No	No	No	No	No
97	No	No	No	No	No	No	No	No	No
98	No	No	No	No	No	No	No	No	No
99	No	No	No	No	No	No	No	No	No
100	No	No	No	No	No	No	No	No	No
101	No	No	Yes	No	No	Yes	No	No	No
102	No	No	Yes	No	Yes	No	No	No	No
103	No	No	No	No	No	No	No	No	No
104	Yes	Yes	No	Yes	Yes	Yes	No	No	No
105	No	No	No	No	No	No	No	No	No
106	No	No	No	No	No	No	No	No	No
107	No	No	No	No	No	No	No	No	No
108	Yes	No	No	Yes	Yes	No	No	No	No
109	No	No	No	No	No	No	No	No	No
110	No	No	No	No	No	No	No	No	No
111	No	No	No	No	No	No	No	No	No
94	No	No	No	No	No	No	No	No	No

Table 6 Obstacles to getting earlier care

ID	Did not know the location of clinic/hospital	Clinic/hospital far away from home	No transport	Long clinic waiting time	Too busy with work	Had many family problems	Financial constraint
1	No	No	No	No	No	No	No
2	No	No	No	Yes	Yes	No	No
3	No	No	No	No	No	No	Yes
4	No	No	No	No	No	No	No
5	No	No	No	Yes	No	No	No
6	No	No	Yes	No	Yes	No	No
7	No	No	No	No	Yes	No	No
8	No	No	No	No	Yes	No	No
9	No	No	No	No	Yes	No	Yes
10	No	Yes	No	No	No	No	No
11	No	No	No	No	No	No	No
12	No	No	No	No	No	No	No
13	Yes	No	No	Yes	No	No	No
14	No	No	No	No	No	No	No
15	No	No	No	No	No	No	No
16	No	No	No	Yes	Yes	No	No
17	No	No	No	No	Yes	No	No
18	No	No	No	No	Yes	No	No
19	No	No	No	No	No	Yes	No
20	No	No	No	No	No	No	No
21	No	No	No	Yes	Yes	No	No
22	No	No	No	No	Yes	No	No
23	No	No	No	No	No	No	No
24	No	No	No	No	Yes	No	No
25	No	No	No	Yes	No	No	No
26	No	No	No	No	No	No	No
27	No	No	No	No	No	No	No
28	No	No	No	No	No	No	No
29	No	No	No	No	No	No	No
30	No	No	No	No	No	No	No
31	No	No	No	No	No	No	No
33	No	No	No	No	No	No	No
34	No	No	No	No	Yes	No	No
35	No	No	No	No	No	Yes	No
36	Yes	No	No	Yes	No	No	No
37	No	No	No	No	No	No	No
38	No	No	No	Yes	Yes	No	Yes
39	No	No	No	No	No	No	No
40	No	No	No	No	No	No	No
41	No	No	No	Yes	No	No	No
43	No	No	No	No	No	No	No
44	No	No	No	No	No	No	No
45	No	No	No	No	No	Yes	Yes
46	No	No	No	No	No	No	No
47	No	No	No	No	No	No	Yes
48	No	No	No	No	No	No	No
49	No	Yes	No	No	No	Yes	Yes
50	No	Yes	Yes	Yes	Yes	Yes	Yes
51	No	No	No	No	No	No	No
52	No	No	No	No	Yes	No	No
53	No	No	No	No	No	No	No
54	No	No	No	Yes	Yes	No	No
55	No	No	No	No	No	No	No
56	No	No	No	No	No	No	No
57	No	No	No	No	No	No	No
58	No	No	No	No	No	No	No
59	No	No	No	Yes	No	No	No
60	No	No	No	No	No	No	No
61	No	No	No	No	Yes	No	No
62	Yes	No	Yes	No	Yes	No	No
63	No	No	No	No	No	No	No
64	No	No	No	No	No	No	No
65	No	No	No	No	No	No	No
66	No	No	No	Yes	Yes	Yes	No
67	No	No	No	No	No	No	No
68	No	No	No	No	No	No	No
42	No	No	No	No	No	No	No
69	No	No	No	No	No	No	No
71	No	Yes	No	No	No	No	No
72	No	No	No	No	No	No	No
83	No	No	No	No	No	No	No
84	No	Yes	Yes	No	No	No	Yes
85	No	No	No	Yes	Yes	No	Yes
86	No	No	No	No	No	No	No
87	No	No	No	No	No	No	No
88	No	No	No	No	No	No	No
89	No	No	No	No	No	No	No
91	No	No	No	No	No	Yes	Yes
92	No	No	No	No	No	No	No
93	No	No	No	No	No	No	No
95	No	No	No	No	No	No	No
96	No	No	No	No	No	No	No
70	No	No	No	No	No	No	No
73	No	No	No	No	No	No	No
74	No	No	No	No	No	No	No
75	No	No	No	No	No	No	No
78	No	No	No	Yes	Yes	Yes	No
80	No	No	No	No	No	No	No
81	No	No	No	No	No	No	No
82	No	No	No	No	No	No	No
97	No	No	No	No	No	No	No
98	No	No	No	No	Yes	No	Yes
99	No	No	No	No	No	No	No
100	No	Yes	No	No	Yes	No	No
101	Yes	No	No	Yes	No	No	No
102	No	No	No	No	No	No	No
103	No	No	No	No	No	No	No
104	No	No	No	No	No	No	No
105	No	No	No	No	No	No	No
106	No	No	No	Yes	No	No	No
107	No	No	No	No	Yes	No	No
108	Yes	Yes	Yes	No	Yes	No	Yes
109	No	No	No	No	No	No	No
110	No	No	No	No	No	No	No
111	No	No	No	No	No	No	No
94	No	No	No	No	No	No	No
